# Sleep targets highly connected global and local nodes to aid consolidation of learned graph networks

**DOI:** 10.1038/s41598-022-17747-2

**Published:** 2022-09-05

**Authors:** G. B. Feld, M. Bernard, A. B. Rawson, H. J. Spiers

**Affiliations:** 1 Division of Psychology and Language Science, Department of Experimental Psychology, Institute of Behavioural Neuroscience, University College London, London, UK; 2 Clinical Psychology, Central Institute of Mental Health, University of Heidelberg, J5, 68159 Mannheim, Germany; 3 Department of Addiction Behavior and Addiction Medicine, Central Institute of Mental Health, Medical Faculty Mannheim, Heidelberg University, Mannheim, Germany; 4 Department of Psychiatry and Psychotherapy, Central Institute of Mental Health, Medical Faculty Mannheim, Heidelberg University, Mannheim, Germany; 5 Department of Psychology, Heidelberg University, Heidelberg, Germany; 6 Aging and Cognition Research Group, German Center for Neurodegenerative Diseases (DZNE), Magdeburg, Germany; 7 Wellcome Trust Centre for Integrative Neuroimaging (WIN-FMRIB), University of Oxford, Oxford, UK

**Keywords:** Sleep, Human behaviour

## Abstract

Much of our long-term knowledge is organised in complex networks. Sleep is thought to be critical for abstracting knowledge and enhancing important item memory for long-term retention. Thus, sleep should aid the development of memory for networks and the abstraction of their structure for efficient storage. However, this remains unknown because past sleep studies have focused on discrete items. Here we explored the impact of sleep (night-sleep/day-wake within-subject paradigm with 25 male participants) on memory for graph-networks where some items were important due to dense local connections (degree centrality) or, independently, important due to greater global connections (closeness/betweenness centrality). A network of 27 planets (nodes) sparsely interconnected by 36 teleporters (edges) was learned via discrete associations without explicit indication of any network structure. Despite equivalent exposure to all connections in the network, we found that memory for the links between items with high local connectivity or high global connectivity were better retained after sleep. These results highlight that sleep has the capacity for strengthening both global and local structure from the world and abstracting over multiple experiences to efficiently form internal networks of knowledge.

## Introduction

Sleep has been shown to support the consolidation of declarative memories^1–3^. A night of sleep will tend to enhance memories compared to a similar period of wakefulness during the day^4–6^). The main mechanism driving this consolidation is thought to rely on the repeated reactivation of recently encoded memories during sleep^7–9^. Over time, the reactivation of overlapping information leads to memory abstraction such that some of the detail is lost and the gist of an experience or the centrally important information is retained^10^. Sleep is thought to be particularly important for the extraction of such gist and the building of schemas^11,12^. Consistent with this, important items encoded before sleep have been shown to be more enhanced by sleep^13–17^. In addition, memory strength and item difficulty affect how much memory is boosted by sleep with low strength and high difficulty items profiting the most^18–20^.

While most studies of sleep have focused on discrete items such as word lists or item pairs^1,2^, most real-world information is interlinked and integrated in networks of knowledge^21^. Thus, it remains unclear how sleep impacts the learning of network structures. It has recently been argued that the hippocampus and parahippocampal structures which support spatial memory and navigation may have evolved in humans to support the learning of knowledge networks more broadly^22–28^. This has extended to concepts in reinforcement learning where optimal policies for learning new information need to be developed^29^.

Recordings from individual cells in the hippocampal-parahippocampal network have provided evidence that neurons with specific tunings support aspects of representing a cognitive map of the environment^24,30,31^. Hippocampal place cells in rats show spatially localised patterns of activity during movement through environments with each place cell being active in different specific regions of an environment^32^. Collectively they provide a unique code for each location encountered in the environment. During periods of sleep and immobility, subpopulations of place cells tend to re-activate, with the order of the cells active ‘replaying’ the sequence of locations visited in an environment previously^33,34^. Such replay appears to preserve the topological structure of the environment, with sequences of replay along routes in a Y-shaped maze consistent with the physical connections within the environment^35^. This suggests that during sleep, hippocampal networks will replay the various paths experienced during the awake state preserving the structure and may replay intersections or paths that are more frequently encountered if replay is linked to the amount of exposure. For example, passing through a central node many times while exploring a network of paths would lead to reactivations passing through that node many more times than other regions.

Using a film simulation of a complex network of recently learned city streets it has been possible to examine evoked hippocampal responses to street networks when navigating^36^. When entering new street junctions, if the new street contained more local streets to choose from (higher degree centrality) then posterior hippocampal activity increased, but if the options decreased (e.g., a dead end) then posterior hippocampal activity declined. While posterior hippocampal activity responded to local connectivity, the anterior hippocampus responded to changes in the globally connectivity (closeness centrality). Its activity increased when entering a more globally connected street in the network and decreased when entering less globally connected streets. Further, a study that trained participants to find routes through a small network of streets in a virtual reality environment showed that increased hippocampal activity during sleep was related to improved performance after sleep^37^. The same group later demonstrated that sleep can restructure navigational behaviour to become less reliant on a spatial hippocampus dependent strategy and engage an additional striatal stimulus response based strategy^38^. Together these studies suggest that the day after learning a street network the hippocampus is able to track the connectivity in a network during navigation and that navigational memories are reactivated and transformed during sleep. We are currently only beginning to understand how information networks and their topological structure are learned and consolidated in the gap between learning and navigation.

Recently a number of studies have begun to explore how graph structures may be learned^39,40^. However, most of these studies only tracked memory for a short period of time or only investigated learning^41–44^. In contrast, a recent study asked participants to learn structured information according to a graph and retrieve it 24 h later in an MRI scanner^45^. Neuronal activity measured in the entorhinal cortex tracked the distance between items within the learned graph. Another interesting study (however with a short retention interval ranging minutes) investigated how local connectivity, i.e., community structure, affects statistical learning and could show that participants are sensitive to this type of topology inasmuch as they identified edges connecting local communities as natural breaking points^44^. However, to our knowledge there has been no research on the impact of local and global connectivity (i.e., degree centrality and closeness centrality) on information processing and memory acquisition in the long-term. Nor have studies examined how sleep may impact learning networks, where theories emphasize the importance of extracting the gist from experience, which arguably would relate to the connectivity of nodes in a network.

**Figure 1:**
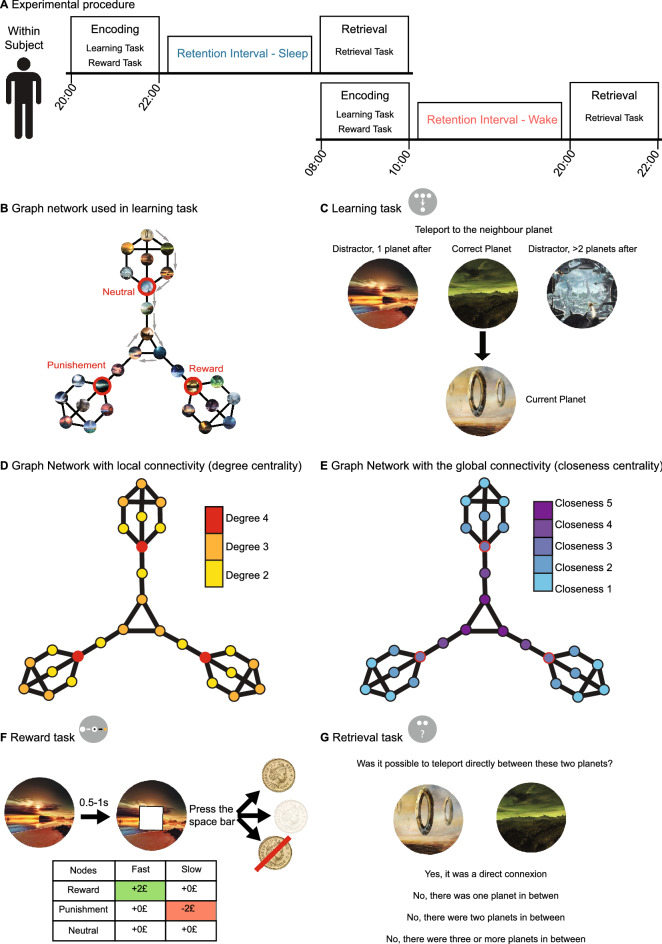
Experimental procedure and task description. (**A**) In our within-subject design participants took part in two identical experimental sessions with parallel versions of the task and retention intervals containing either sleep or wakefulness. The learning phase started at 8:00 a.m. (or p.m.) and participants performed a learning task (see **C**) and a reward task (see **F**). After the 10-h retention interval, participants came back to the lab to complete the retrieval phase (see **G**). After 1 week, participants returned to perform the other experimental session with the remaining retention interval. (**B**) Representation of the undirected graph structure composed of 36 edges (black lines) linking 27 nodes (circles with examples of stimuli presented during the experience). Red nodes represent reinforced nodes, either positive (reward), negative (punishment) or neutral. This image was never shown to the participants, but determined the structure of the learned associations. (**C**) During learning, participants saw one planet at the bottom depicting their current position on the graph and three planets to choose from displayed at the top. After choosing, the choice was marked but only the correct planet (i.e., the one connected to the bottom planet according to the graph) moved down to replace the bottom planet. Then a new set of three planets appeared at the top prompting a new choice. Participants performed eight such choices (transitions) taking an eight-step route through the graph (an example route is indicated by arrows in (**B**). After each route, they received feedback on their performance and a new route started at a pseudo-random location on the graph. Participants performed 81 routes in total. (**D**) To construct the graph the graph-theoretical parameters of degree centrality (number of direct connections for a given node) and (**E**) closeness centrality (inversely proportional to the number of steps to every other node on the graph) were orthogonalized (i.e., allowing to independently asses their effect on retention) and a three-fold symmetry was pursued (to enable equal positions for the reinforced nodes). (**F**) During the reward task, participants were shown a planet representing one of the three reinforced nodes (reward, punishment and neutral). After 0.5–1 s a white square appeared on top of the picture. Dependent on the participants pressing the spacebar quickly enough, they were shown the outcomes at the bottom (for details see “Reward task” below). (**G**) During the retrieval task, participants were shown two planets taken pseudo-randomly from the graph network and had to answer whether they were directly connected or whether one, two or three and more planets were in between.

Here, we examine how sleep during retention affects associations that were learned using an explicit graph-learning task with a topology that allowed us to disentangle contributions of local and global connectivity. We expected (1) that weaker/more difficult associations would be improved more by sleep during the retention interval (as has been demonstrated elsewhere^18–20^, meaning that greater distance between nodes would predict a greater benefit from sleep during the retention interval, (2) that important information would be improved more by sleep during the retention interval, such that high centrality (local or global connectivity) would predict a greater benefit from sleep during the retention interval. In our design edges connected to high and low centrality nodes did not systematically differ in exposure during learning as we carefully and pseudorandomly chose routes. Since relevance has been shown to generally enhance the sleep effect^17^, we did not expect there to be any differences between nodes of high global or local connectivity in their susceptibility to sleep-dependent memory effects but that they would be equally enhanced by sleep compared to nodes of low global or local connectivity. In addition, we contrasted centrality derived relevance with more classical reinforcement related relevance, by associating some of the nodes with monetary reward and punishment. To prevent confounds of centrality and reinforcement related effects we constructed a graph with three-fold rotational symmetry and placed the rewarded, punished and neutral nodes at symmetrical positions within the graph (see Fig. 1). Of note, in this initial experiment we only included men to increase the likelihood of finding the hypothesised differences between sleep and wake. In future studies, we plan to generalise these finding to women (see “Methods” section for details).

Box 1. Graphs We refer to graphs in their mathematical form to describe connections (edges) between instances (nodes) and not, as more commonly used, as a form of data presentation. Graphs are part of our everyday life and are most easily visualized as networks of connections. For example, the connections of the London Underground can be described using a graph. Here, the different stations are the nodes and the connections are the edges, i.e., the node Russel Square is connected to the node Euston via the node King’s Cross and two edges. The degree centrality (local connectivity) and closeness centrality (global connectivity) of a given node signify its relevance in the network. For example Oxford Circus has a high degree centrality as it is directly connected to 6 other stations, but it also has a high closeness centrality as you can travel to any other node relatively quickly (i.e., using few edges).

## Methods

### Participants

Twenty-five healthy young men aged between 18 and 30 years (24.20 ± 3.53) took part in the study. Women have been shown to vary regarding learning and memory in general and sleep-dependent consolidation specifically across the menstrual cycle^46^ and the high time intensity of sleep research constrains the possibility of increasing power by increasing the sample size^47,48^. Therefore, men were chosen for this study to reduce the variance in the sample, which optimised statistical power under these constraints^49^. Notably, simply adding twenty-five women to our sample would not enable us to robustly detect differences between men and women since this would be a between-within as well as a higher order interaction effect that would most likely require many more participants to detect^50^. Participants were non-smokers, fluent in English, not currently under medication and did not have any physical or mental disorders. They all reported having a regular sleep schedule, going to bed before midnight (11:18 p.m. ± 47 min) and waking up before 8:00 a.m. (7:41 a.m. ± 47 min). In addition, participants did not work during night shifts and were not diagnosed with sleep disorders and they did not travel across time zones. Finally, they did not report any stressful events such as exams or deadlines before or during the experiment. The experiment was approved by the UCL ethics committee (ID number: 8951/002) and all research was performed in accordance with relevant guidelines and regulations. Written informed consent was obtained from each participant before starting the experiment. Participants were compensated financially for their participation.

### Design and procedure

The study was performed in a balanced, within-subject design where participants came for two sessions separated by at least 7 days (8.44 ± 2.98). Each session was composed of a learning and a retrieval phase with a retention interval of 10 h between the two phases. At the end of the learning phase, participants were asked to avoid learning new information or studying and to not rehearse the information they had learned. Two conditions, sleep and wake, were tested in the experiment for each participant. In the wake condition, participants came at 8 a.m. to complete the learning phase and returned at 8 p.m. for the retrieval phase. In the sleep condition, participants arrived at 8 p.m. and returned the following day at 8 a.m. (see Fig. 1A). The sequence of conditions was counterbalanced across participants.

The experiment was divided into two phases, in the first, the learning phase, participants completed the learning and the reward tasks and in the second, the retrieval phase, they completed the recall task (for details, see task description below). At the end of each phase, control measures were taken. Mood of the participants was assessed by asking them to fill in the Positive And Negative Affective Scale (PANAS^51^) their subjective sleepiness was measured with the Stanford Sleepiness Scale (SSS^52^) and reaction speed (1/reaction time, i.e., the reciprocal of reaction time), an objective measure of vigilance, was obtained from the 5-min version of the Psychomotor Vigilance Task (PVT^53^). We chose the 5-min version over longer versions of the PVT as it reduces the burden on participants and provides sufficient power to detect effects of sleep-deprivation, if reaction speed (1/reaction time) is used as outcome variable^54^. Additionally, at the end of each retrieval phase, participants performed a word generation task to assess their ability to retrieve highly consolidated information^55^. Word generation is a standard measure used in many sleep and memory studies and has also been used successfully to detect long-term memory deficits in very mild cases of dementia^56^.

Finally, at the end of the second retrieval phase, participants filled in the Santa Barbara Sense-Of-Direction scale (SBSOD^57^) asking questions about spatial and navigational abilities and completed the Navigational Strategies Questionnaire (NSQ^58^) asking questions about their experiences with navigation and their navigation strategy.

### Graph structure

A graph consisting of 27 nodes was constructed (a representation of the graph can be seen in Fig. 1B) and pictures were assigned to each node (unique landscapes of extraterrestrial planets). The number of nodes was chosen to enable effective encoding within the 1.5 h of the learning phase (as determined by pilot participants’ performance). The graph contained 36 edges that connected the nodes. During construction, the graph-theoretical parameters of closeness centrality and degree centrality were orthogonalized and a three-fold symmetry was pursued (Fig. 1D,E). In addition, three nodes corresponding to the red nodes in Fig. 1B were selected to be reinforced. The nodes were either positively reinforced (reward node), negatively reinforced (punishment node) or not reinforced (neutral node). The reinforcements were associated during the reward task (for details, see task description below). This graph was never shown to the participants during the experiment and participants were not explicitly told about an underlying structure of the learning task.

### Learning task

The learning task was gamified to optimise participants’ motivation during learning and retrieval (see Supplementary Methods for details). Briefly, the task was embedded in a storyline of humankind on the brink of extinction on earth and participants explored planets to find a new home for humans to live. Our piloting demonstrated that participants’ motivation, especially during the 1.5 h of the learning session, greatly benefitted from this approach, which enabled us to use a somewhat larger graph. To familiarise the participant with the stimuli, each planet was shown in the middle of the screen with its name under it for 2 s with an inter-stimulus-interval of 0.5 s. Next, the participants learnt the graph structure by performing 81 routes of 8 transitions length between the planets of the graph. An overview of the task can be seen in Fig. 1. For each transition, the participants were asked to identify the neighbour of the current planet that was presented with its name at the bottom of the screen (i.e., the planet connected by a single edge) while being shown three potential planets with their names at the top of the screen. One option was the correct planet (one of the 2–4 connected planets) and the two other options were incorrect (i.e., not directly connected). Of the two wrong choices, one of the planets had a distance of two edges, i.e. there was one planet between the current planet and the incorrect choice, and the other had a distance of three or more edges, i.e., there were at least two planets in between. The wrong choices were chosen randomly from all planets qualifying the distance argument and only the shortest distance was considered relevant for this choice. During each transition, participants had a maximum of 10 s to choose the correct transition. If they did not answer within the time limit, the choice was considered incorrect and the next trial was presented.

### Reward task

The reward task was constructed to be an adaptation of the monetary incentive delay task that robustly activates reward areas^59^. During the task, participants saw the three reinforced nodes mentioned above. During 180 trials, a fixation cross appeared in the middle of the screen for 250 ms followed by one of the three planets representing the nodes for 2 s. After 0.5 to 1 s, a white square appeared and the participants needed to press the spacebar as fast as possible (pressing before the square was shown was considered a miss). Depending on their RT and the planet presented, the participants obtained a different monetary outcome (Fig. 1F). For the reward planet, if the participants were fast enough, they got + 2£, but if they were not fast enough, they got 0£. For the punishment planet, being fast enough let them earn 0£, but being too slow made them lose − 2£. Finally, for the neutral planet participants got 0£ whether they were fast or not. Their wins and losses accumulated resembling the amount they would receive for performing the reward task.

### Retrieval task

The retrieval task was divided into three parts. In the first part, we presented two planets (without their names) side-by-side and asked participants to decide if the planets were directly connected during learning, or if there were one, two, or three or more planets in between (Fig. 1G). This was done for all the possible combinations of the 27 planets, therefore the participants were presented with 351 trials (if participants chose completely randomly they would be correct in 25% of cases). In the second part, we asked the participants about the names of the planets. Participants performed 27 trials, one for each planet, and were asked which of four possible names was correct. The three incorrect names were chosen to be from planets that were one, two or more edges away, respectively. In the last part, the participant identified the contingencies learned in the reward task again. This means they were shown the different planets used for the reward task and asked to identify the neutral, reward and punishment planet, respectively.

### Data reduction and statistical analysis

The data of 6 participants were excluded from the analysis. Four participants had a learning performance with an accuracy lower than 0.5 and two participants had a high learning performance, accuracy above 0.8, but a low retrieval performance, less than 0.4 (see Supplementary Fig. 5). We excluded participants with low learning performance (i.e., not substantially above chance level of 33%) as they would have little to no knowledge about the underlying graph network structure, which would be required for the node topology taking effect. We excluded participants with high learning but low retrieval scores, as these participants likely did not learn the higher order associations required for high performance at retrieval and thus would likewise not be able to rely on knowledge about the graph. These planned exclusions occured before comparing the retention score, our main outcome variable, between sleep and wakefulness. In addition, only node pairs with distance 1 to 4 were analysed since the retrieval tested participants’ knowledge of the graph structure only to distance 4 at maximum. Data reduction was performed in Matlab 2018a and the statistical analysis depended on R studio (Version 1.0.143). The analysis relied mainly on repeated measures ANOVA, paired t-tests, pearson correlations and regression with linear modelling. Details regarding the data reduction and statistical analysis can be found in the Supplementary Methods.

## Results

### Retention performance

**Figure 2:**
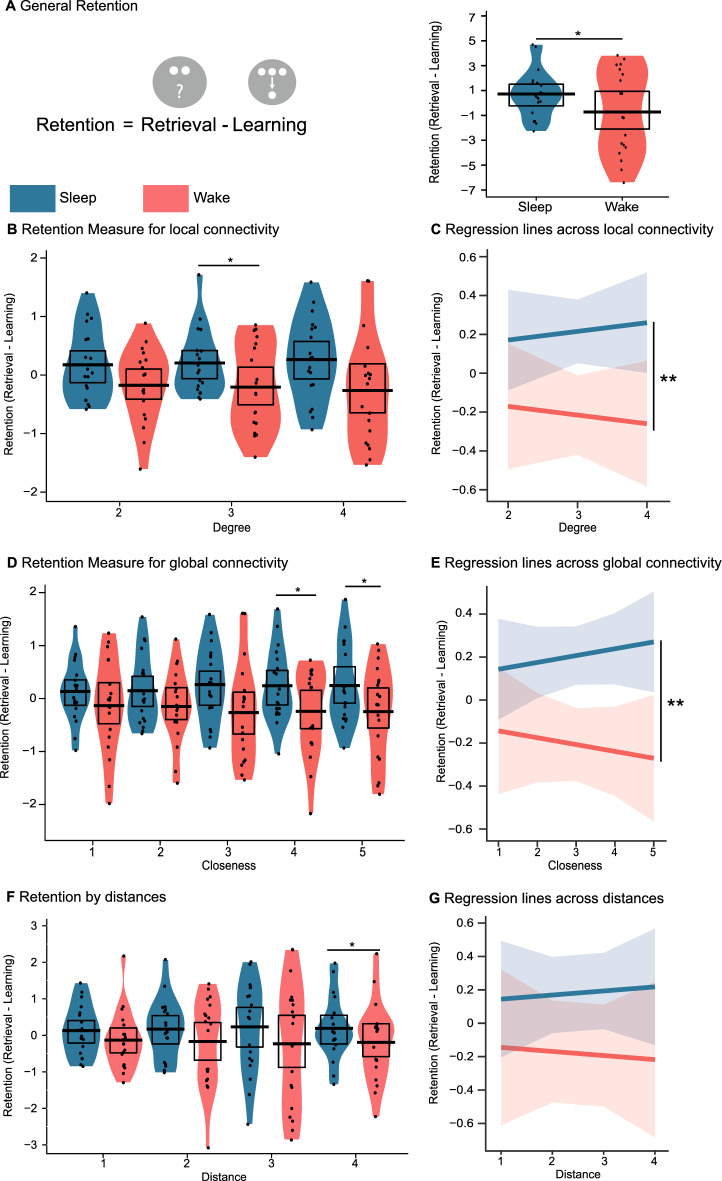
Retention performance. (**A**) Left, the retention measure was calculated by substracting the learning performance from retrieval performance. Details can be found in Supplementary Fig. 4. Right, mean overall retention performance for the sleep (blue) and the wake (red) condition. (**B**) Mean retention performance and (**C**) regression model for the different distances within the graph, (**D**) and (**E**) for the different levels of degree centrality of the nodes and (**F**) and (**G**) for the different levels of closeness centrality of the nodes. For the violin plots, the black dots represent the individual performance, the black bar represents the mean across participants, the black rectangle shows the 95% of a Bayesian highest density interval and the coloured shape displays the smoothed density. *p < 0.05, **p < 0.01.

A retention measure was created by subtracting the learning performance from the retrieval results, i.e., how much information the participant retained during the retention period (Fig. 2A; for more details see the Supplementary Methods section and Supplementary Fig. 4, but note performance is normalized to a mean of 0 and a standard deviation of 1 before substraction). Importantly, the learning performance and retrieval performance were highly correlated (r = 0.88 and r = 0.87 for sleep and wake, respectively), which indicates that the retention measure is a valid outcome measure. When collapsing all distance information, we found that participants retained more information from learning to retrieval in the sleep condition than in the wake condition (t(18) = 2.13; p = 0.048). An ANOVA across distances, confirmed this main effect of interval (F(1,18) = 4.52; p = 0.048). When analysing the distances individually we found that for distance 4 the sleep condition also showed better retention (t(18) = 2.29; p = 0.035, Fig. 2F). However, no such difference was found for distances 1 (t(18) = 1.01; p = 0.327), 2 (t(18) = 1.33; p = 0.201) and 3 (t(18) = 1.37; p = 0.188). Using linear regression modelling, which in contrast to ANOVA allowed us to take into account the order of the distance predictor, the distances per interval revealed a difference of intercept between the sleep and wake conditions, which can be interpreted in the same way as a main effect in an ANOVA (F(1,18) = − 3.99; p = 0.016). However, this analysis did not reveal a difference in the slopes, which can be interpreted in the same way as the distance by interval interaction in an ANOVA (F(1,18) = − 2.22; p = 0.091, Fig. 2G). Of note, since neither the ANOVA nor the regression models found evidence for a distance by interval interaction, only the main effect of sleep should be interpreted and post-hoc pairwise comparisons are only included for completeness.

Regarding the local connectivity (degree centrality), participants retained more information during the sleep interval (F(1,18) = 4.71; p = 0.044). This effect was stronger for nodes of degree centrality 3 (t(18) = 2.15; p = 0.046) compared to degree 2 (t(18) = 2.01; p = 0.060) or 4 (t(18) = 1.89; p = 0.075, Fig. 2B). The regression analysis confirmed the main effect of sleep (difference in intercept: F(1,18) = − 4.86; p = 0.039) and indicated that higher degree centrality was associated with an increased benefit from sleeping during retention (difference in slope: F(1,18) = − 8.26; p = 0.015, Fig. 2C).

Similar results were found for global connectivity (closeness centrality) since participants again performed better across the sleep retention interval (F(1,18) = 5.26; p = 0.035), which was mirrored by a sleep benefit for closeness centrality of 4 (t(18) = 2.27; p = 0.036) and 5 (t(18) = 2.90; p = 0.001) but not for closeness 1 (t(18) = 1.10; p = 0.285), 2 (t(18) = 1.41; p = 0.174) or 3 (t(18) = 1.89; p = 0.075) (Fig. 2D). Again the regression analysis showed that participants performed better across sleep (difference in intercepts: F(1, 18) = − 3.79; p = 0.009) and that a higher degree centrality increased the effect of sleep (difference in slopes: F(1, 18) = − 3.59; p = 0.012, Fig. 2E).

### Learning and retrieval performance

**Figure 3:**
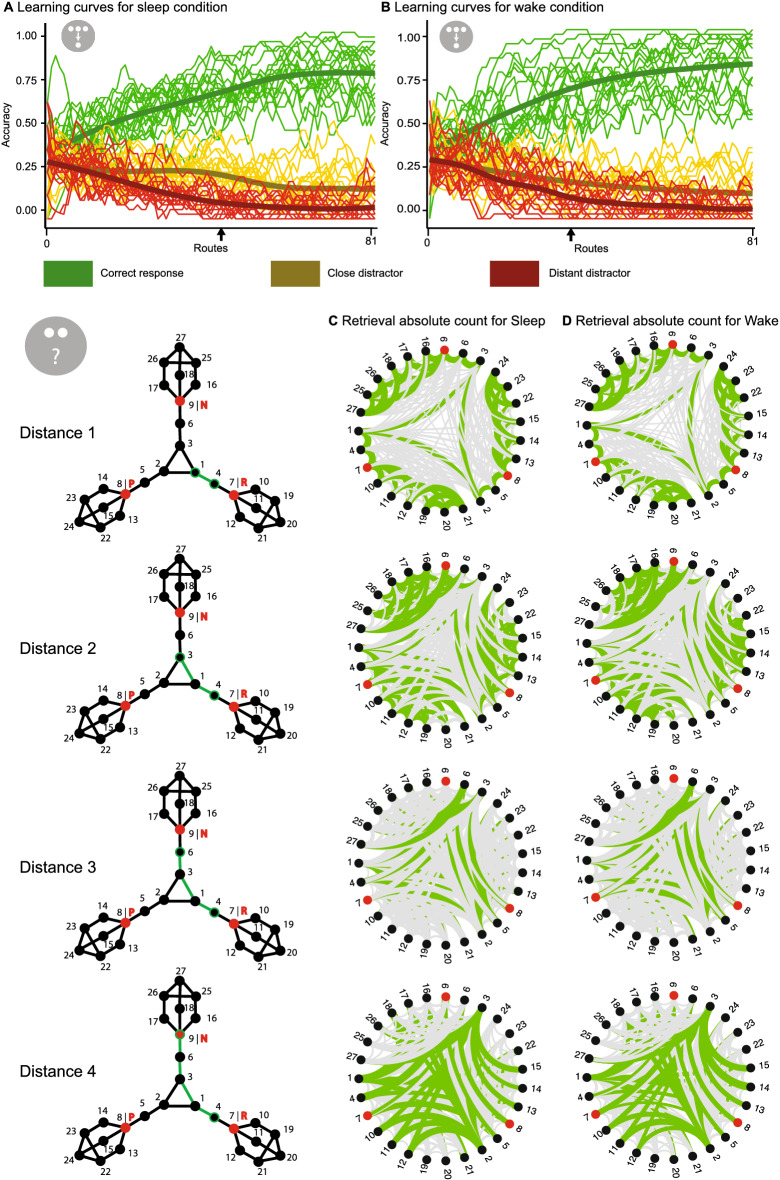
Participants’ raw learning and retrieval data. (**A**) Learning curve across the 81 routes for the sleep and (**B**) the wake condition. Mean (thick line) and individual responses (thin lines) for correct response (green), the close distractor (yellow) and the distant distractor (red) smoothed by a five point moving average. The black arrow indicates when participants’ performance was significantly biased by the graph structure (i.e., accuracy of the close and distant distractor started to differ). (**C**) Retrieval task data for each distance (rows) in the sleep and (**D**) the wake condition. On the left the graph structure and an example (in green) is shown for each distance, which is defined by the number of edges between the pair of nodes tested. Within the circles, green lines represent correct connections and grey lines correspond to incorrect connections at that distance, whereas line thickness depicts how many participants gave the respective answer.

For the learning results, descriptively overall learning performance (calculated by averaging the encoding results over the four distances, see Supplementary Methods) was greater for the wake than for the sleep condition, but this difference was not statistically significant (t(18) = − 2.09; p = 0.051, Supplementary Fig. 1B). However, when viewing only first order connections (as learned during the task) and dividing the learning task into thirds, no main effect of sleep or wake was evident when analysing the three thirds in an ANOVA (F(1,18) = 2.66; p = 0.120) or for the last third in an individual t-test (t(18) = − 1.09; p = 0.289, Supplementary Fig. 1A). Although the participants increased their learning performance across the thirds (F(1, 18) = 124.74; p < 0.001). The individual learning curves of participants are shown in Fig. 3A,B for both conditions.

Regarding local connectivity, an ANOVA showed that higher degree centrality was associated with better learning (F(1, 18) = 44.55; p < 0.001) but no effect of intervals (F(1, 18) = 4.17; p = 0.056) nor an interaction was found (F(1, 18) = 0.63; p = 0.540) (Supplementary Fig. 2A). For global connectivity, similar results were found, i.e., participants learned nodes with higher closeness centrality better (F(1, 18) = 33.01; p < 0.001) (Supplementary Fig. 2C) but there was no effect for the retention interval (F(1, 18) = 4.33; p = 0.052) and no interaction (F(1, 18) = 1.56; p = 0.195). Similarly, no difference in intercept and slopes were found for the linear model analysis.

Regarding the general retrieval performance (calculated by the hit rates for each distance, see Supplementary Methods), participants were better at correctly identifying closer pairs than more distant ones (F(1,18) = 7.46; p < 0.001), but there was no main effect of the interval (F(1,18) = 0.37; p = 0.551) nor an interaction effect (F(1,18) = 0.13; p = 0.939) (Supplementary Fig. 1C). A visualisation of the raw retrieval data can also be found in Fig. 3C,D. No statistical difference was found for local connectivity (F(1, 18) = 2.88; p = 0.069, Supplementary Fig. 2B) but global connectivity showed a main effect of the centrality (F(1, 18) = 4.34; p = 0.003) (Supplementary Fig. 2D). Similar to learning, no statistical differences were found for the linear model analysis. Finally, participants were not able to remember planet name associations better across the sleep than the wake condition (t(18) = 0.89; p = 0.384).

### Reinforcement

**Figure 4:**
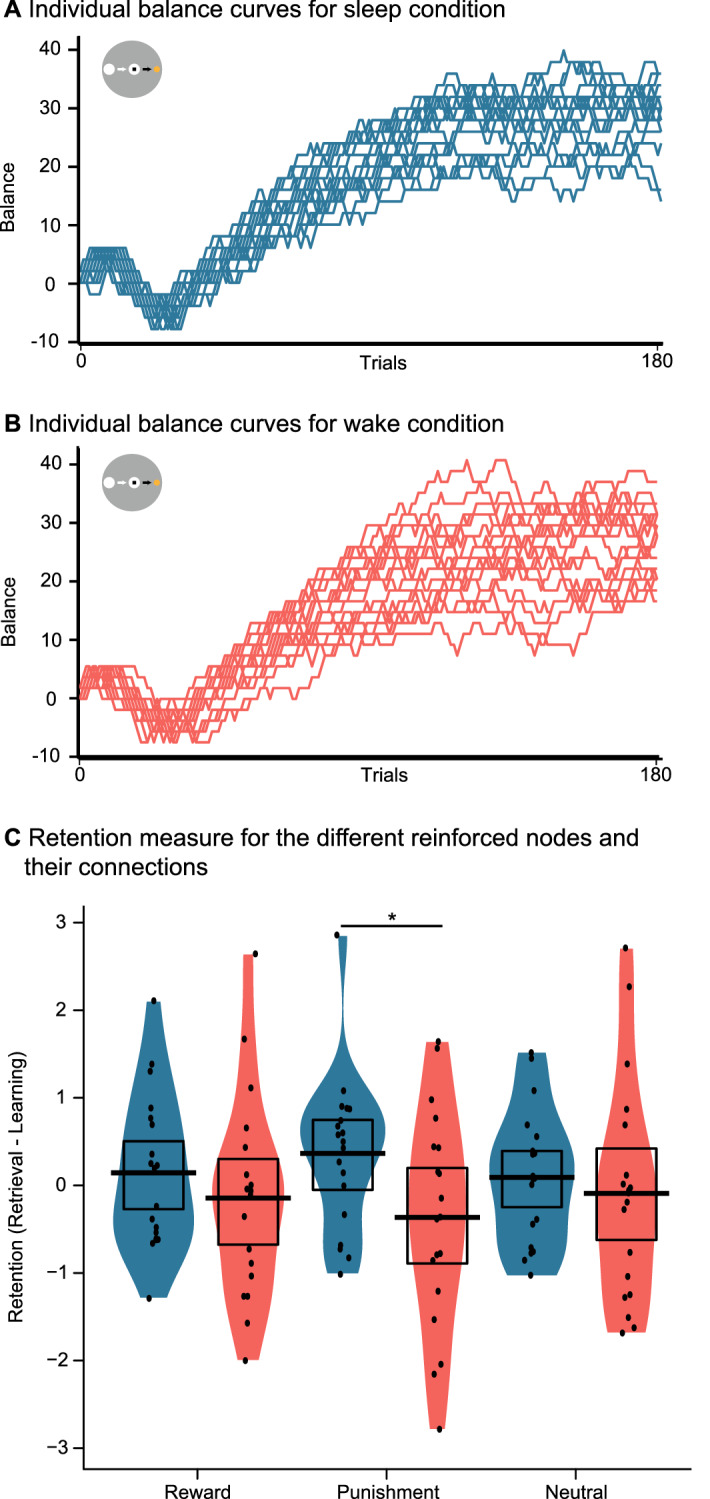
Reinforced nodes results. (**A**) Individual monetary balance curves during the reward task in the sleep and (**B**) the wake condition. Participants earned the amount reached at the end of the task. (**C**) Retention performance for the reinforced nodes. The black dots, bar and rectangle represent the individual performances, the mean and the 95% of a Bayesian highest density interval, respectively. The coloured shape shows the smoothed density. *p < 0.05.

Individual balance curves (the amount of money given to participants, see “Methods” section), for the two conditions can be found in Fig. 4A,B. Regarding the retention measure, an ANOVA found no influence of sleep or wake (F(1, 18) = 2.33; p = 0.144) nor was there an effect of reinforcement (F(1,18) = 0; p = 1) or an interaction of the two (F(1,18) = 1.12; p = 0.337) (Fig. 4C). However, exploratory paired t-tests found that for the punishment node participants performed better in the sleep condition (t(18) = 2.10; p = 0.049) but not for the reward (t(18) = 0.92; p = 0.368) and neutral nodes (t(18) = 0.49; p = 0.629). Note that participants could explicitly recall the reward contingencies after the reinforcement task, which was verified by the investigator by showing the three planets and asking about the associated monetary outcomes.

### Navigation tests

**Figure 5:**
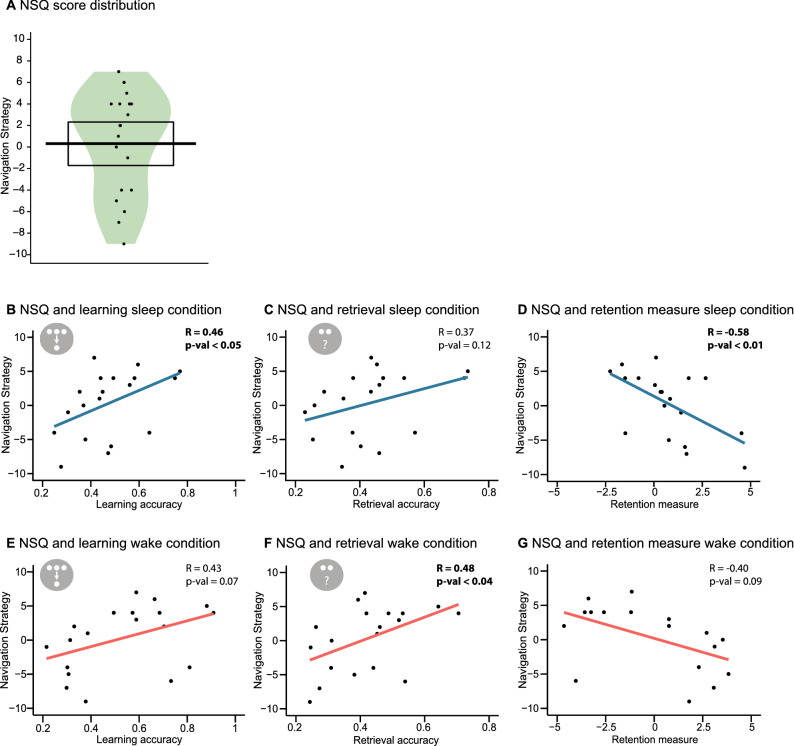
Correlation of the results and the navigation score. (**A**) The Navigation Strategies Questionnaire (NSQ) score, the black dots, bar and rectangle represent the individual performances, the mean and the 95% of a Bayesian highest density interval, respectively. The coloured shape shows the smoothed density. (**B**) Relationship of the Navigation Strategies Questionnaire (NSQ) with learning performance, (**C**) retrieval performance and (**D**) retention performance for the sleep condition and with (**E**) learning performance, (**F**) retrieval performance and (**G**) retention performance for the wake condition. Regression lines (blue—sleep, red—wake) and black dots for the individual data points are shown.

A significant correlation was found between the two navigation questionnaires (r = 0.55, p = 0.015). Since the variance of the NSQ was larger, further correlations used this navigation test (Fig. 5). Participants with a higher score in the NSQ have a higher mapping strategy. We found a significant negative relationship between the NSQ and the retention measure for the sleep (r = − 0.58, p = 0.009) but not for the wake condition (r = − 0.40, p = 0.093), no statistical difference was found when comparing the correlation coefficients using Fisher’s Z procedure (Z = − 0.85, p = 0.393). We found a positive relationship between the NSQ and learning performance in the sleep condition (r = 0.46, p = 0.048) and between the NSQ and retrieval performance in the wake condition (r = 0.48, p = 0.036). Correlations for learning performance in the wake condition (r = 0.43, p = 0.066) and retrieval performance in the sleep condition (r = 0.37, p = 0.122) did not reach significance. In general, although some relationships did not reach significance in the sleep or the wake conditions the overall pattern of effects was similar. In addition, no differences were found between the correlation coefficients when comparing learning (Z = 0.23, p = 0.821) or retrieval (Z = − 0.79, p = 0.432) correlations between sleep and wake.

### Control tasks

There was no difference in the long-term retrieval performance (measured with the word generation task) between the sleep and the wake conditions (t(18) = − 0.82; p = 0.423). Also, no statistical difference was found for objective vigilance (reaction speed, i.e., the reciprocal of reaction time, of the PVT), subjective sleepiness (measured by the SSS) or positive or negative affect (measured by PANAS) between the sleep and the wake conditions during learning (PVT: t(18) = 1.02, p = 0.321; SSS: t(18) = − 0.57, p = 0.578; PANAS-positive: t(18) = 1.37; p = 0.188; PANAS-negative: t(18) = 2.08; p = 0.052) or retrieval (PVT: t(18) = 0.66, p = 0.519; SSS: t(18) = − 0.86; p = 0.399; PANAS-positive: t(18) = 0.67; p = 0.509; PANAS-negative: t(18) = − 0.33; p = 0.742). Descriptive statistics can be found in Table 1.

**Table 1:** Data for control variables.

	Sleep		Wake	
	Mean	SD	Mean	SD
Word generation	41.16	2.73	42.79	2.95
**PANAS—positive**				
Learning phase	30.16	1.52	28.36	2.21
Retrieval phase	28.17	1.86	26.89	1.52
**PANAS—negative**				
Learning phase	14.10	0.77	12.58	0.58
Retrieval phase	11.84	0.59	12.00	0.55
**PVT**				
Learning phase	3.13	0.08	3.08	0.07
Retrieval phase	3.17	0.08	3.14	0.06
**SSS**				
Learning phase	2.63	0.23	2.79	0.29
Retrieval phase	2.42	0.18	2.69	0.22

## Discussion

Here, we investigated the impact of sleep on consolidating learned topological networks, which varied across them in global and local connectivity of nodes. We found both connections to globally and locally highly relevant nodes were preferentially enhanced by sleep. This was despite equal exposure to all the connections in the network during learning. By contrast, sleep had no impact on the enhancement of nodes made salient by monetary reinforcement. We discuss how these results help advance our understanding of how representations of learned graph networks are affected by offline processing, models of sleep and consolidation and implications for understanding offline replay in hippocampal networks.

The current study presents a novel associative learning task, where information was learned according to a graph network. We found that during learning, the graph influenced behaviour beyond first level associations inasmuch as close distractors (distractors that were only one edge away from being a direct connection) were more frequently chosen than distant distractors (distractors that were at least two edges away from being a direct connection) when participants made errors. Although sleep enhanced memory retention per se, its effect was not enhanced by increased distance between the nodes, as might have been expected due to sleep preferentially enhancing items with low association and high difficulty^18–20^. Sleep did however specifically enhance items that were more relevant for navigating the network as items of high local and of high global connectivity showed a stronger sleep effect. This graded effect of topological relevance may be related to findings of graded reward effects on memory within a maze^60^. In this study, participants explored a maze by uncovering cards laid out in a 2-d grid and received a high or a low reward after a certain amount of cards. The reward effect was higher the closer a card was to the final card that was uncovered. Although we found a similar graded effect of topological relevance, we found no effect of monetary reinforcement applied to a subset of nodes. This may likewise be explained by a spread of reward across the network, if one assumes that our network was too small. We chose the size of our network after extensive piloting so that it could be learned to about 80% correct within 1.5 h. Using a larger network or maybe even two networks with different reinforcement procedures may prove more fruitful.

Sleep has been suggested to enhance the abstraction of gist from episodes^10^. Such gist abstraction may become more important, when larger networks are learned, as participants will struggle to keep all connections in memory. Representing the network at different scales would then allow for more efficient memory processing. Integrating very large networks may occur over several nights of sleep, as has been shown for other gist abstraction processes^11^. Once a large network has been built, it may prove that new items can be added to this schema much faster and with less reliance on the hippocampus^61,62^. In fact, sleep may no longer be required to consolidate such memories since encoding may circumvent the hippocampus directly integrating into the neocortical knowledge network^63^. However, based on past work showing this rapid learning of new nodes to networks^61,62^, we would predict that, after adding new nodes to our network, this newly encoded information would require less sleep consolidation to stabilize as long as the global topology is minimally affected and therefore systems consolidation would be completed much faster. This could be studied in our paradigm by adding nodes that do or do not strongly influence topology. For example, providing a single shortcut between very distant parts of the network could radically change the global connectivity of nodes and potentially drive more extended consolidation during sleep.

One prediction from theories highlighting the importance of global gist extraction and schema development^10,64^ is that predominantly the globally important information would be prioritised over the local important information. We did not find this was the case, since high local connectivity also impacted consolidation during sleep. In future research, it would be interesting to explore memory after several days to observe whether global and local information is lost or retained at the same rate. Testing other network structures would also help explore whether the local and global effects are additive, in that are nodes with both high local and global centrality doubly enhanced by sleep?

According to one perspective, hippocampal replay during sleep has been considered to be closely aligned with prior experience, so that sequences of place cells evident during learning would emerge again during subsequent sleep^34^. Reward has been shown to influence replay of place cells and enhancing reward increases the frequency of replay events in brief rest intervals during learning^65^. Another study showed that enhancing dopaminergic modulation within the hippocampus enhances replay frequency during post encoding rest^15^. An alternative perspective has recently suggested that in the absence of reward replay events may represent random samples from available trajectories through space^66^. The enhancement of globally relevant nodes in our network could be explained by either account. Either global relevance was inferred already during wake encoding and enhanced replay of those nodes during sleep by a synaptic tagging mechanism^67^, or the structure of the network may have biased replay that occurs in form of random walks on the graph to emphasize nodes with high betweenness centrality. As betweenness and closeness centrality were highly correlated in our graph, we cannot at present distinguish which of the two metrics might impact consolidation during sleep. With a larger network it would be possible to dissociate closeness centrality and betweenness centrality (see Fig. 1 of^36^). If replay takes a random walk through the network it might specifically enhance regions of high betweenness centrality but not closeness centrality. However, it may be that replay prioritizes important structures to be learned^68^, which recent evidence supports^69^.

Here we found preliminary evidence (small sample correlation) that participants who show efficient graph learning tend to have a higher self-reported estimated tendency to navigate via maps and thinking in terms of maps to navigate, based on the NSQ. This dovetails with recent evidence that people who make better inferences about the structure of graph networks show more model-based planning on a multi-step planning task^70^. Future research with large-scale cohorts online^71^ would be a useful way to explore the robustness of such correlations and what other moderating factors may influence these relationships. In this vein, it would also be important to understand whether the negative relationship between NSQ scores and retention performance constitutes a true relationship, or is driven by the positive relationship of both learning and retrieval performance.

There are several limitations to the current work that should be considered. We chose a day wake vs. night sleep comparison, which comes with the down side of a circadian confound. Choosing a sleep deprivation vs. normal sleep comparison instead, has been argued to induce the problem of unspecific effects on cognition at retrieval^72^. This can be ameliorated by adding recovery sleep, which, however, has been shown to reduce the beneficial effect of sleep by putatively allowing postponed sleep dependent consolidation^73^. We weighed these possibilities in our decision and statistically there was no difference in performance between the conditions where learning took place in the morning vs. the evening. However, descriptively learning performance was higher in the morning and we cannot rule out that there may be a circadian effect on learning that we did not detect due to sample size limitations. Although, our analysis relied on difference scores, which take potential differences at baseline into account, there remains the possibility of ceiling effects in the wake condition. We also employed a different retrieval than learning procedure. We did this, as during retrieval, we were interested in the higher order associations that go beyond the first order associations that were learned. Adding an immediate retrieval after learning would have confounded the experiment, since then it would have been unclear whether sleep-dependent consolidation acted on the learned information or the immediately retrieved information. Although we took great care when choosing the graph topology for this experiment to orthogonalize the centrality measures of interest (closeness and degree centrality), it was beyond the scope of this research to test different graph topologies, which therefore limits the generalizability of our findings. Additionally, while the three-fold symmetry of our graph was used to limit confounds of the reinforced nodes on the centrality related findings, we cannot completely rule out that our results would be different for graphs that do not feature any reinforced nodes. Surprisingly, we found that retention performance in the second experimental session was reduced rather than being increased, which would usually be expected for repeating a memory task. A speculative but reasonable explanation for this may be that the abstracted graph information from the first session interfered with retention of the graph structure in the second experimental session. Since interference has been argued to interact with the sleep effect^74–76^, it would be interesting to systematically investigate this effect in the future. Alternatively, although we took care not to inform the participants about the graph structure explicitly, the retrieval procedure likely enabled them to guess that there was structure beyond the paired associates, which could be harnessed to strategically improve memory during the second experimental session. However, since retention performance was reduced in the second experimental session, this seems unlikely. We also did not find that the planet name associations were affected, which would be expected given the broad support for word-pair memory and other associative memory benefitting from sleep during the retention interval (see^77^ for a meta-analysis). This may have been due to the arbitrariness of the names or due to other components of the task being more relevant. Our choice to ask for planets four or more edges away but only analysing up to four edges away put an unnecessary burden on participants and may have introduced a bias, inasmuch as, most answers would be 4 or more. Future research should only collect data for nodes up to four steps away. Larger future studies could also attempt to shed light on the difference between people who could and couldn’t learn the task (4 participants were unable to learn) as well as those people who could learn the task, but were unable to retrieve higher order multiple step associations (2 participants).

In conclusion, we find that local and global aspects of connections between individual items of a declarative associative memory task determine access to sleep-dependent memory consolidation. This approach has the potential to explore in more detail how replay influences the knowledge structure of declarative memory.

## Supplementary Information

Supplementary Information.
