# Supplementary material for: Sleep targets highly connected global and local nodes to aid consolidation of learned graph networks
Source: Sci Rep. 2022 Sep 5;12:15086. doi: 10.1038/s41598-022-17747-2 (PMC9445065; doi:10.1038/s41598-022-17747-2)
Supplement: Supplementary file 1 — Supplementary Information. [file 41598_2022_17747_MOESM1_ESM.pdf]

Sleep targets highly connected global and local nodes to aid consolidation of learned graph networks

Feld GB, Bernard M, Rawson AB & Spiers HJ

Supplementary Methods

General procedures

All the learning tasks were programmed and executed on Matlab2016a with the package Psychtoolbox-3. Matlab was running on Dell OPTIPLEX 990 with Windows 7 Enterprise with a Dell P2011H monitor (resolution of 1600x900). Up to four participants were run at one time in individual cubicles.

Two storylines were created and were told using texts during the instructions, as well as, videos before and after the tasks, to keep the participants interested and focused. The first storyline, corresponding to the first session, emphasized that Earth will be destroyed and participants are sent into a new galaxy to explore it. The second storyline, related to the second session, told that there are strange sounds and radio wave frequencies that are being emitted from a neighbouring galaxy. This time, participants must explore this galaxy to lead to further understanding of what these sounds may mean. Videos that helped build this storyline can be found in the supplementary methods. The stimuli were presented on a screen that contained elements of a videogame to further gamify the experience (see below for details).

Both sessions used the same protocol and participants learnt the same graph structure but with a different set of stimuli (i.e. pictures) for each session. In addition, each participant received a uniquely randomised version of the task with different stimuli, planets, names and routes. For each memory task, instructions and examples were presented at the beginning and participants had the opportunity to ask any questions after the instructions.

Learning task

After each route that consisted of 8 transitions, the participant received feedback of their performance, i.e., they were shown up to three stars with a written sentiment (0 stars – Try harder, 1 star – Well done, 2 stars – Superb, 3 stars – Amazing). Furthermore, participants could keep track of their progress by consulting a bar at the right side of the screen that was updated after each transition. Moreover, a rank system was created and participants were promoted after completing every 27 routes ('Novice'; 'Lieutenant Jr.'; 'Lieutenant'; 'Captain'; 'Major'; 'Colonel'; 'Commander'). An "energy level monitor" at the bottom was filled up to 1500 at the beginning of the experiment. For additional motivation, the participants lost 2 units each time they got an incorrect answer whereas only 1 unit was lost for a correct choice. This bar was related to the storyline as depleting too much energy would produce negative consequences for humankind.

Reward task

To learn these associations between planets and gain/loss, the criterion, i.e., the maximum time for the reaction that participants needed to match, changed throughout the task. At the beginning, the criterion was slow so participants would accumulate 6£, even if they were reacting rather slowly, since the task was very easy. Then, the criterion was sped up so participants mostly lost money and reached -6£ as the task became very hard. For the rest of the task, a linear function was applied to the RT data of the last twenty trials to set the criterion that scaled with the amount of accumulated money, if the participant was lower than 24£ in their balance, the task was easier, and if they had more it was harder and the difference in criterion was proportional to the difference in money. This procedure ensured that participants received approximately the same total of money after the experiment and experienced the different contingencies sufficiently frequently. After finishing 180 trials of the task, the participants were asked to indicate which planets predicted which outcome, by showing them the planets consecutively and asking whether it was possible (1/ "I could win money." 2/ "I could NOT lose or win money." 3/ "I could lose money.").

Similar as in the learning task, participants could keep track of their progress on a bar on the right-hand side. Moreover, they could also see in real-time what their current balance was at the bottom of the screen.

Retrieval task

Similar to the learning task, a bar on the right side tracked the progress of the task. Furthermore, a bar on the bottom side, the “Map Quality Monitor”, revealed the participant’s performance during the task, the bar was updated after each feedback slide. Participants obtained more stars by being correct for the distance 1 compared to the other distance (2, 3, 4 and plus) as they were less numerous. This feedback system used stars similar to the learning task and was used for the two first parts (0 stars – Try harder, 1 star – Well done, 2 stars – Superb, 3 stars – Amazing). The feedback appeared every 9 trials for the first part (corresponding to the pictures associations), and every 3 trials for the second part (corresponding to the planet names).

Control tasks

The PANAS contains 10 positive (e.g.; interested) and 10 negatives adjective (e.g.; scared) describing the participant’s current mood with a scale ranging from 1= “not at all” to 5= “very much”. The SSS asks the participant to choose from 1 = “Feeling active, vital, alert, or wide awake” to 8 = “Asleep”. During the PVT, participants were facing a red counter on a black screen and were asked to press the spacebar as soon as the clock would start to count. Pressing the space would stop the counter and show to the participant their reaction time. Then, the inverse of the mean of their reaction speed (i.e.: 1/mean RT) was calculated for each phase. The word generation instructed to write as many words as possible in two minutes for either letter cues (p or m) or a category (occupations or hobbies).

Pilot testing

Before obtaining the final paradigm described above, 3 pilot participants were run only on the learning task and another 6 pilot participants underwent the complete procedure of one experimental session. All the gamification aspects were added during the development of the paradigm in tight dialogue between the researchers and the participants to ensure high

motivation and clear task instructions. The analysis strategy of the results is based mainly on the experience made during the pilot study.

An analysis of the pilot data suggested that the task could be learnt at a 70-80% accuracy level and retrieved with an overall hit rate of 60% (which was significantly higher than the overall false alarm rate). Although the learning and the retrieval accuracy were encouraging regarding feasibility of the paradigm, we decided to change the retrieval task in two distinct ways to improve on this. Initially, participants were asked if the two nodes presented were neighbours (i.e., “Was it possible to teleport between these two planets” [during learning]) and the participant could answer how sure they were from a scale from 1 to 4 (1/ “Yes, I am sure this was possible.” 2/ “Yes, but I am not sure this was possible.” 3/ “No, but I am not sure this was impossible.” 4/ “No, I am sure this was impossible.”). We changed this to the procedure asking for a distance judgement described above that allows a finer grained analysis of the graph representation. The same confidence scale (1/ “Yes, I am sure it was.” 2/ “Yes, but I am not sure it was.” 3/ “No, but I am not sure it was not.” 4/ “No, I am sure it was not.”) was initially used for the second part of retrieval that, where we showed planet name pairs and asked whether they belonged together (i.e., “Was this planet named...?”). We changed this design to the four alternatives forced choice procedure described above that reduced the amount of items to 27, as participants had complained about the length of the original task (729 items).

Data reduction and statistical analysis

The retrieval data were analysed by calculating the hit rates (the number of times pairs of a certain distance were correctly identified,divided by all the possible pairs of this distance) for distance 1 to distance 4. Then, a repeated-measure 2 x 4 ANOVA (sum of squares type III) with the factors sleep/wake and the 4 distances was done. In addition, a paired t-test was performed between the sleep and the wake condition on the general retrieval performance calculated by taking the mean over the four distances for each participant.

Data from the learning task was reduced by calculating the mean of the accuracy for each third of the task, i.e., the mean of routes 1-27, 28-54 and 55-81 for each participant and each retention interval. Then, a repeated-measure 2 x 3 ANOVA (sum of squares type III) with

the factors sleep/wake and task third was performed to assess the learning across the task. A paired t-test was completed between the sleep and the wake condition on the last third to further assure comparability. Using the method described below, the learning performance for each distance was used to create a mean for both retention intervals to get the general learning performance. Then, a paired t-test was completed between the two conditions.

To obtain the learning results for each distance, we weighed each edge of the graph to take into account all the learning data. Therefore, for distance 1, the weight for a transition depended on its position in the learning task. For example, if participants saw a transition 8 times, the weight for the first time they saw it would be 1/8, second time would be 2/8 and last time would be 8/8. Next, the weights were multiplied by the accuracy (1/0), summed together and divided by the sum of all the weights in order to give a single accuracy value between 1 and 0 for each transition. This ensured that instances closer to the end of the task were weighted more for learning accuracy than instances at the beginning. For distances 2 to 4, the graph structure was used to calculate performance between two given nodes. Specifically, the weighted accuracies of the distance 1 edges along the path between the two nodes were multiplied. Finally, the weighted performance for each distance was normalized separately by using the mean and standard deviation calculated with the data from both sleep and wake. The retention measure between learning and retrieval was calculated by deducting the normalized learning data from the normalized retrieval performance for each distance (see also Supplementary Figure 5).

Using the retention measure data, a paired t-test was performed between the sleep and the wake condition on the general performance by averaging over the distances. Additionally, a repeated-measure 2 x 4 ANOVA (sum of squares type III) with the factors sleep/wake and the 4 distances was calculated. Single paired t-tests were performed between the retention intervals for each distance. In addition, to account for the order of the distances, a regression line was created for each interval. Then, a linear model was applied to compare if there was a difference of intercept or slopes between the two regression lines. Finally, a

paired t-test was also performed between the first and the second experimental sessions to investigate order effect (t(18)=2.45; p=0.025).

The retention measures for each centrality (i.e. degree and closeness) were computed by averaging the retention measure data between the nodes with the centrality of interest (e.g. all nodes with a value of degree centrality of 3) and all the other nodes of the graph connected to them from distance 1 to 4 (e.g. for distance 1, it includes the neighbouring nodes of each node with a value of degree centrality of 3. Then, the nodes connected with a distance of 2, 3 and 4). Next, a repeated-measure 2 x 3 ANOVA (sum of squares type III) with the factors sleep/wake and the centrality measure was performed. In addition, paired t-tests were performed between the retention intervals for each centrality. Moreover, the method using regressions lines as described for distances above was used for both centrality measures.

The effect of reinforcement was calculated using a similar method to the centrality analysis. For each reinforced node (i.e. reward, punishment and neutral), the retention measure was calculated using the mean between the weighted edge accuracies of the reinforced node of interest (e.g. reward node) and the nodes being on the same arm of the graph. For example, if we use the node as numbered in Figure 3, the nodes we would compare to the neutral node would be 6, 16, 17, 18, 25, 26 and 27 as we are excluding the central nodes (i.e. 1, 2 and 3). Then, one repeated-measure 2 x 3 ANOVA (sum of squares type III) with the factors sleep/wake and the 3 reinforced arms was performed. Finally, several paired t-tests were done between the sleep and the wake condition for each of the reinforced nodes.

The retrieval of planet-name associations was analyzed by summing the correct answers and dividing them by the number of items for each condition. A paired t-test was then performed to compare the two retention intervals.

Regarding the control tests, pen and paper data (SSS, PANAS and Word generation) were transferred into excel files and scored according to the test instructions. A single missing value in the PANAS was replaced by the mean of the items that were not missing within that scale for the participant. For the word generation task, sum scores for the letter and category cues were added together to create an overall score of retention performance for each retrieval

session. The RT data from the PVT were transformed to reaction speed by dividing one by the RT for each trial. Then, the trials were averaged for each condition and participant. For all the control tasks paired t-tests were applied to compare sleep and wake.

The NSQ and SBSOD were scored according to the instructions. A unique missing value resulted in not taking the specific question into account and dividing the final score with the number of questions minus the number of questions missing. Thus, the NSQ gave a mapping tendency score from -14 to 14 and the SBSOD a score from 1 to 7 where the higher the score, the better one's perceived sense of direction. A first pearson correlation compared the scores for both navigation scores. Then, pearson correlations were done between the NSQ scores and both intervals for learning, retrieval and retention measure data.

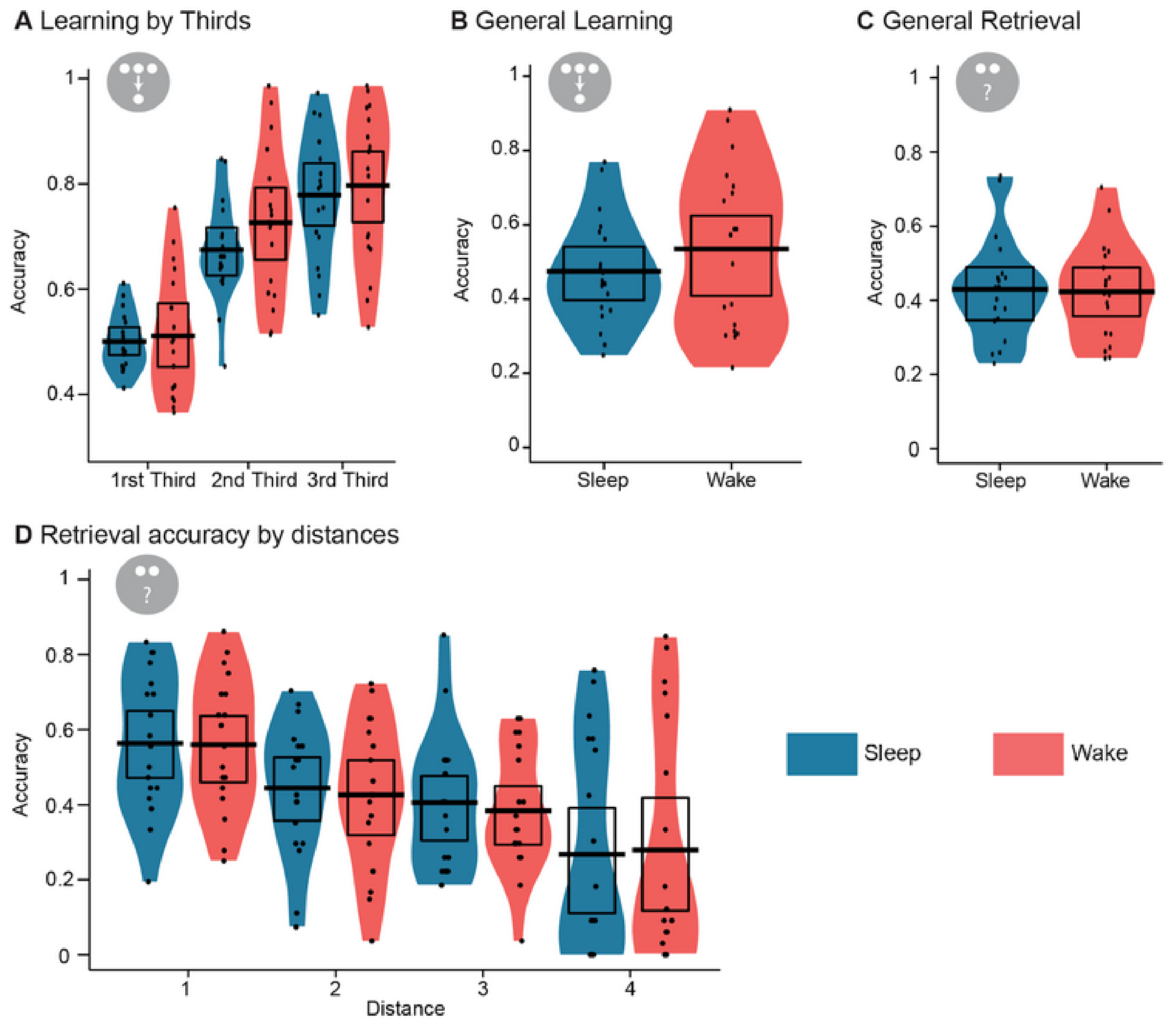

Supplementary Figure 1. Learning and retrieval performance. A) Learning performance (proportion correct) during the learning task for the first, second and last third of the task (27 routes each) and B) for the whole task. C) Retrieval performance for the whole task. D) Retrieval performance for the different distances. The black dots, bar and rectangle represent the individual performances, the mean and the 95% of a Bayesian highest density interval, respectively. The coloured shape around shows the smoothed density.

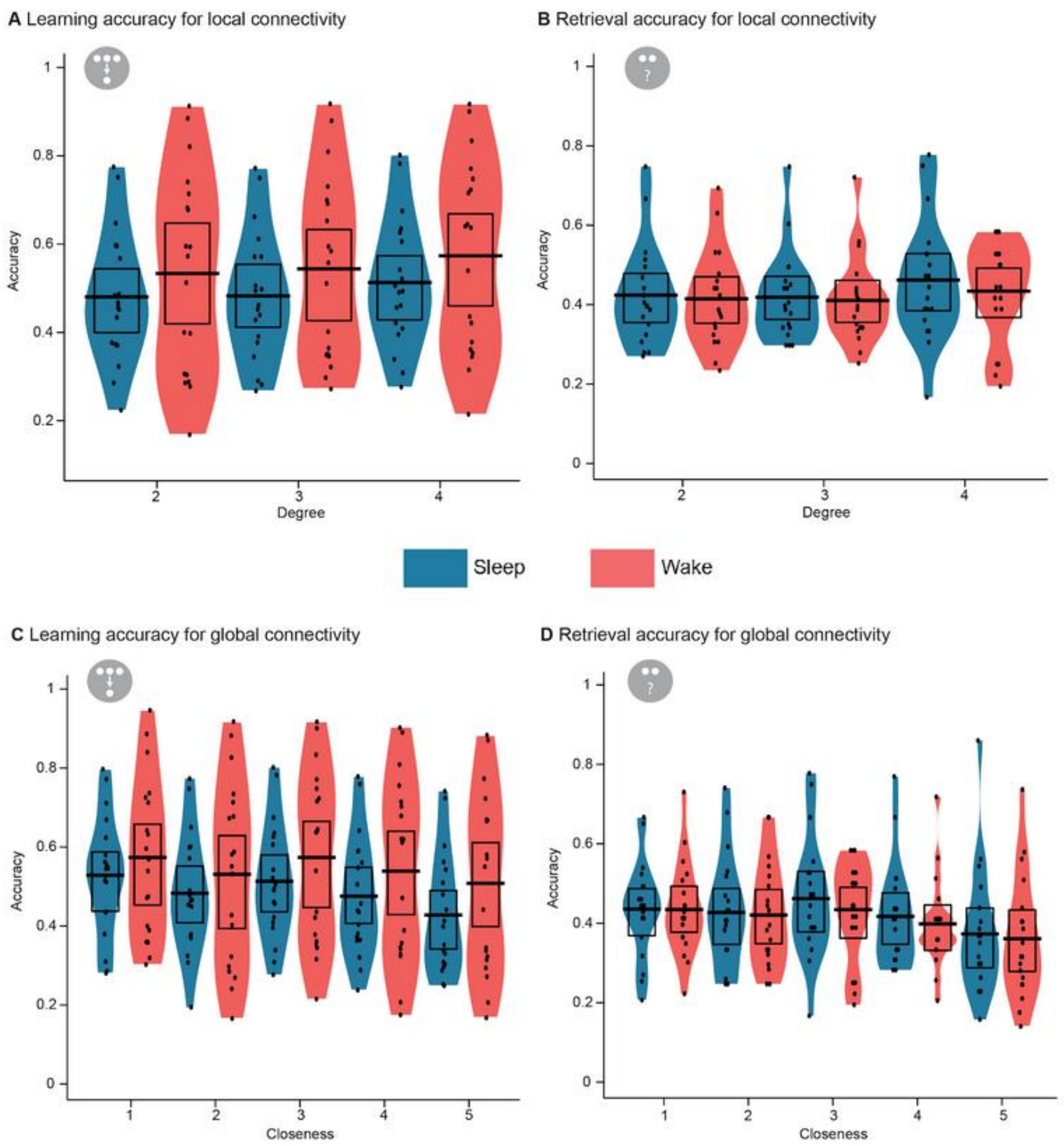

Supplementary Figure 2. Learning and retrieval performance for centrality measures. A) Learning and B) retrieval performance for different levels of degree centrality (local connectivity). C) Learning and (d) retrieval performance for different levels of closeness centrality (global connectivity). The black dots, bar and rectangle represent the individual performances, the mean and the 95% of a Bayesian highest density interval, respectively. The coloured shape around shows the smoothed density.

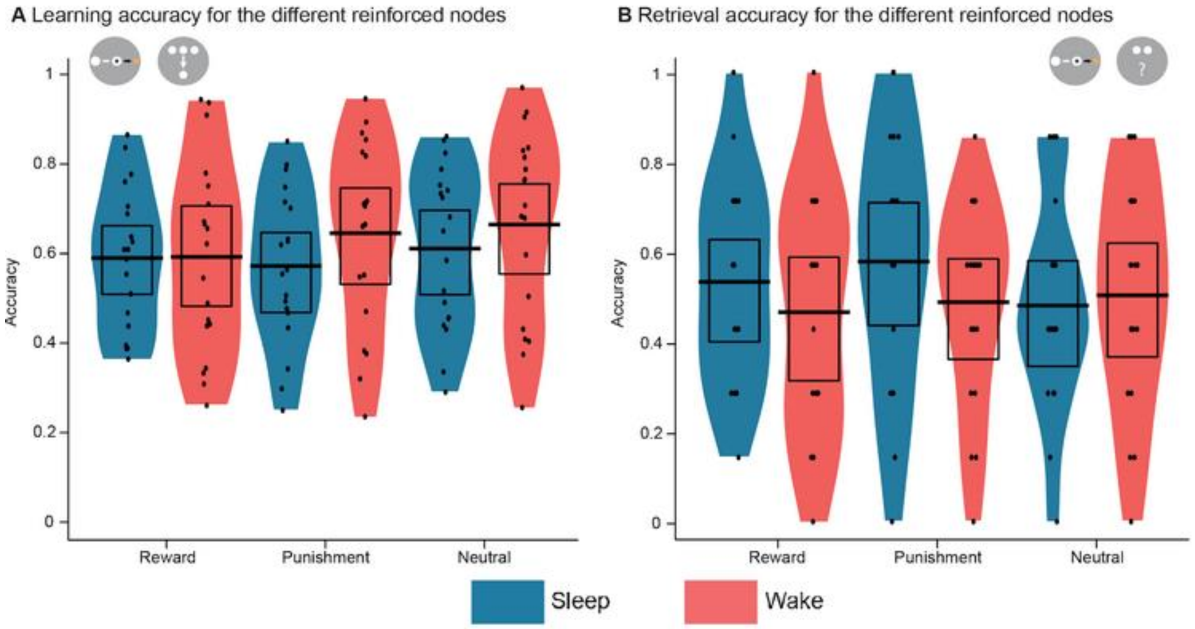

Supplementary Figure 3. Learning and retrieval performance for the reinforced nodes. A) Learning and B) retrieval performance across the three reinforced nodes. The black dots, bar and rectangle represent the individual performances, the mean and the 95% of a Bayesian highest density interval, respectively. The coloured shape around shows the smoothed density.

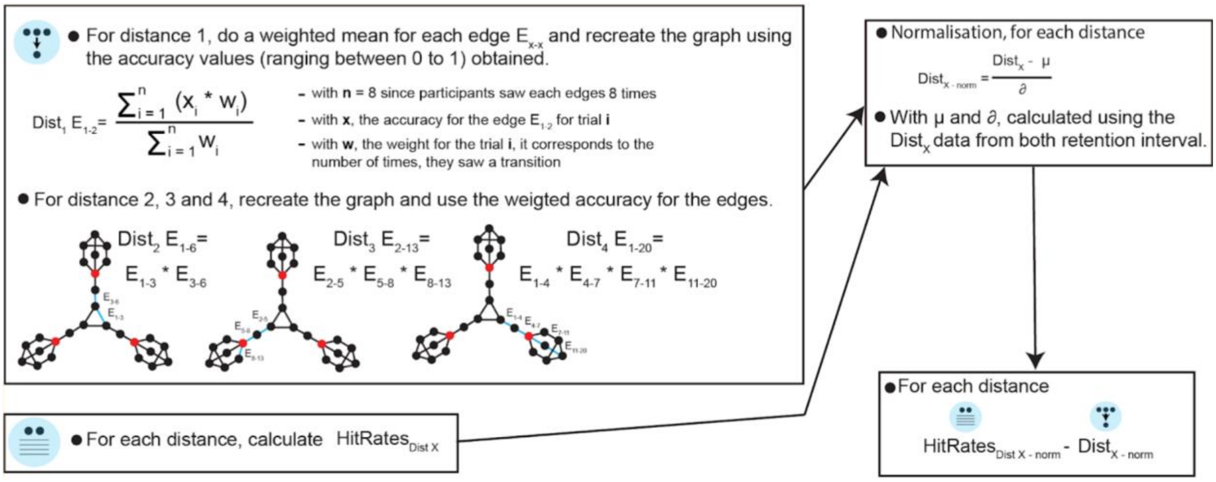

Supplementary Figure 4. Schematic explaining the analysis to obtain the retention measure. During learning, participants saw each transition eight times (n = 8) and the learning performance at distance 1 was calculated as a weighted mean of these repeated trials. To take into account that the later repetitions provide a better estimate of performance they received a higher weight (w). The weights we used were one to eight, so that the accuracy for the first experience of the transition was multiplied by one and the last experience was multiplied by eight. All of these multiplied accuracies were summed and (to receive a weighted mean accuracy with values between zero and one) we then divided this sum by the sum of the weights (1 + 2 + 3 + 4 + 5 + 6 + 7 + 8 = 36). For further distances, the weighted accuracies for distance 1 of the respective transitions were combined by multiplication. For example as shown in the figure, the distance 2 between nodes one and six would be calculated by multiplying the distance 1 accuracy for the transition between one and three with the distance 1 accuracy for the transition between three and six. For the retrieval performance we used the hit rate for each distance. The learning and the retrieval performance for each distance was normalized individually (but jointly for the sleep and wake condition) using a z-transformation, i.e., the mean was subtracted from the measurement and the result was divided by the standard deviation. Finally, we calculated the difference between the normalized learning and retrieval performance. Supplementary figure 8 shows that learning and retrieval performance is highly correlated, which supports the comparability of learning and retrieval performance and calculation of a difference score.

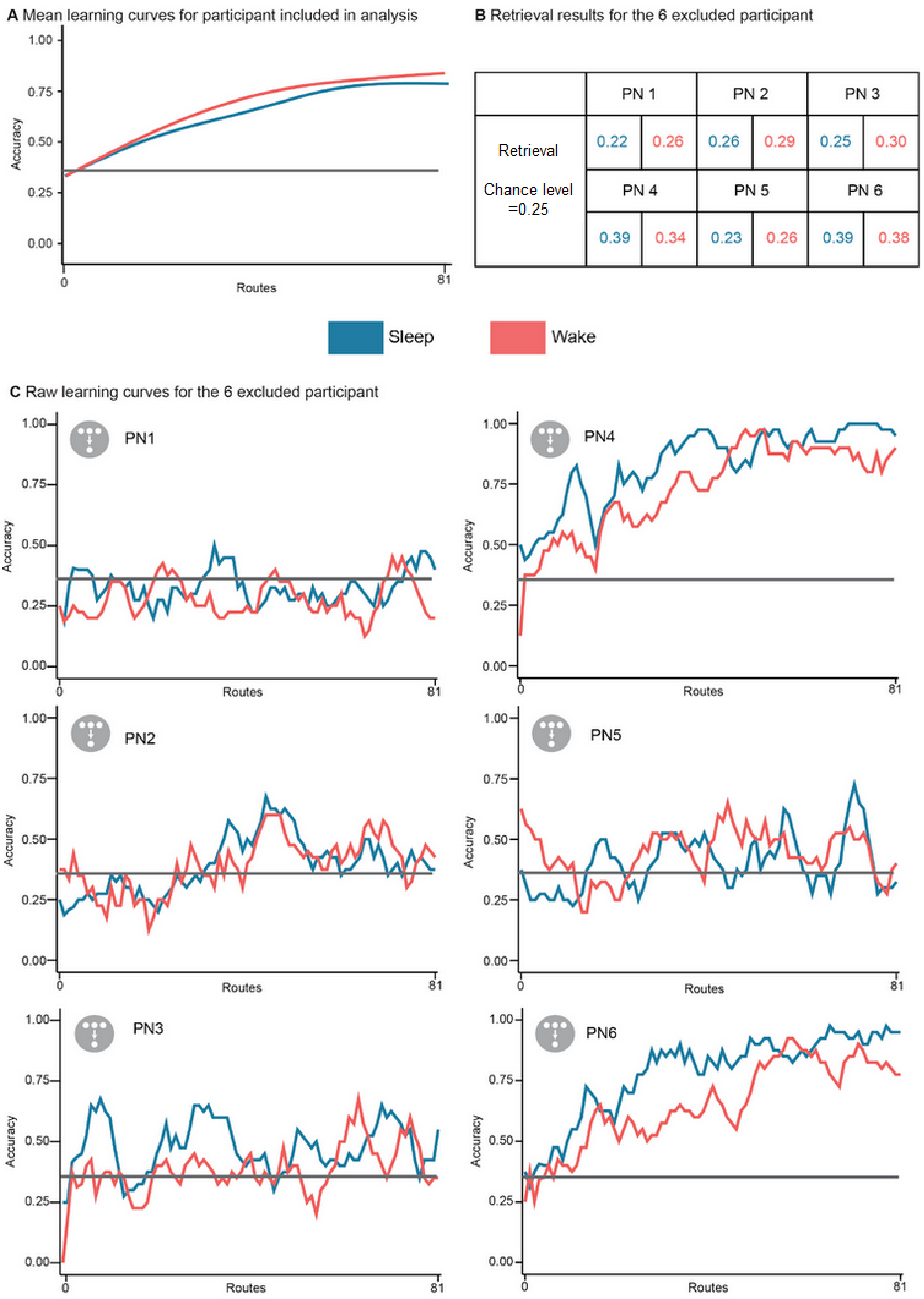

Supplementary Figure 5. Performance of excluded participants. A) Average learning curves for the participant included in the analysis for sleep and wake conditions. B) Retrieval performances of the 6 excluded participants with C) their associated learning curves. Note: P4 and P6 learn but show poor retrieval. The grey bar in the plots represents the chance performance during learning.

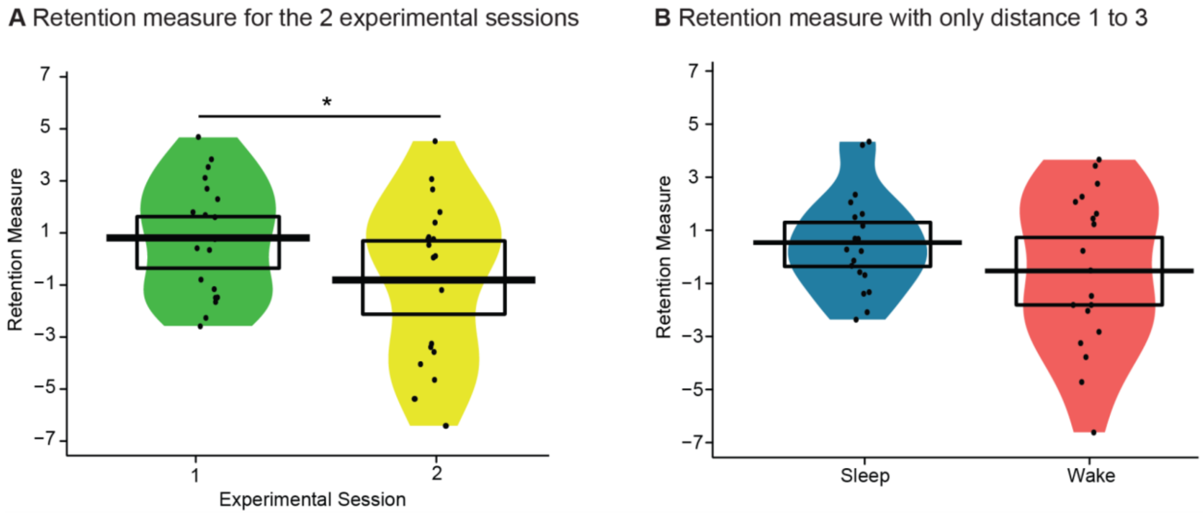

Supplementary Figure 6. A) Retention measure for the first and the second experimental session participant performed (t(18)=2.45; p=0.025). B) Retention measure when only distances between 1 to 3 are included in the analysis (t(18)=1.79; p=0.091). The black dots, bar and rectangle represent the individual performances, the mean and the 95% of a Bayesian highest density interval, respectively. The coloured shape around shows the smoothed density. *p<0.05

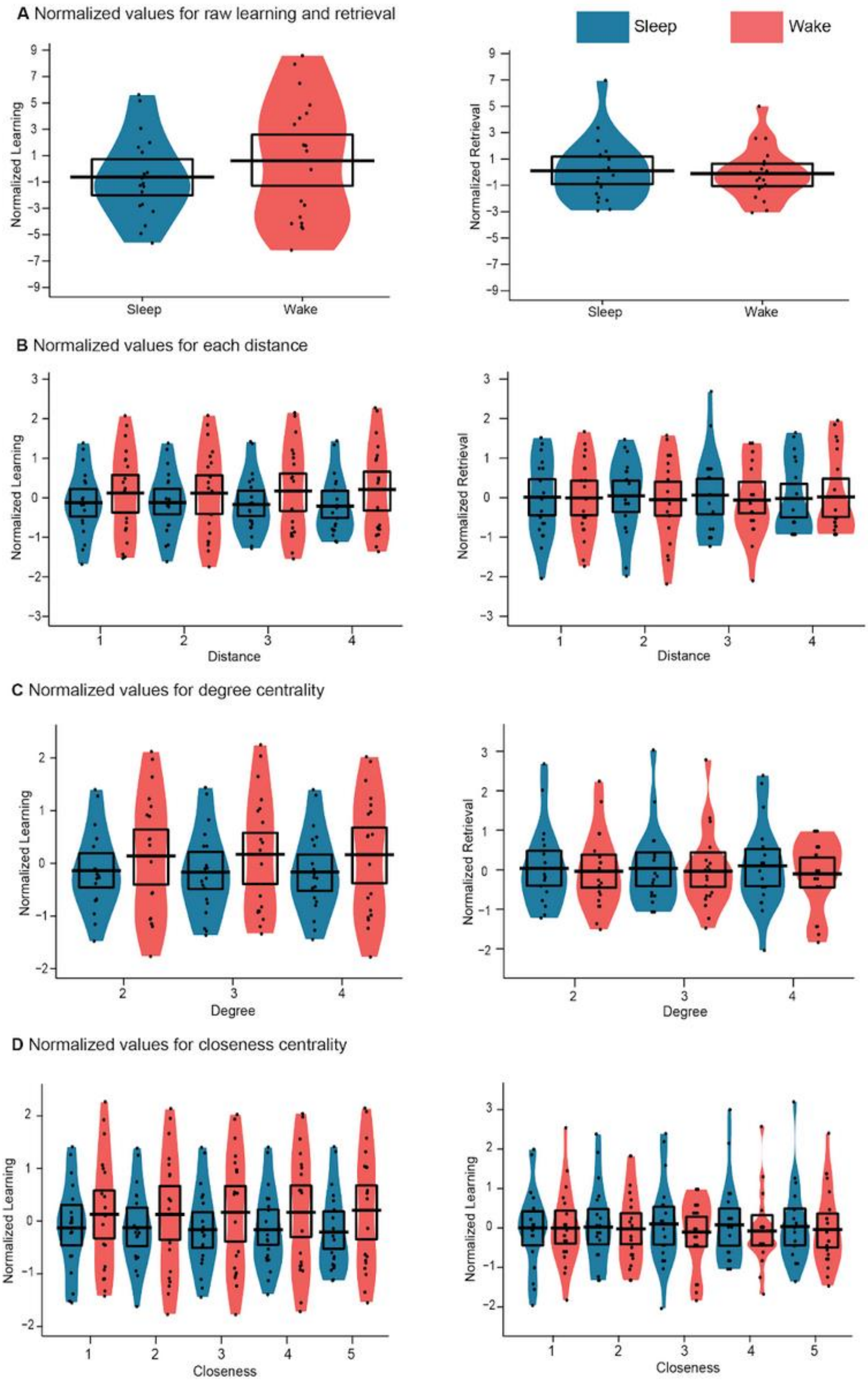
A Normalized values for raw learning and retrieval
Sleep
Wake
Normalized Learning
Normalized Retrieval
Sleep
Wake
B Normalized values for each distance
Normalized Learning
Normalized Retrieval
Distance
C Normalized values for degree centrality
Normalized Learning
Normalized Retrieval
Degree
D Normalized values for closeness centrality
Normalized Learning
Closeness

Supplementary Figure 7. Normalized values before the retention measure calculation for learning (left column) and retrieval (right column) for A) overall performance B) each distance C) degree centrality D) closeness centrality. The black dots, bar and rectangle represent the individual performances, the mean and the 95% of a Bayesian highest density interval, respectively. The coloured shape around shows the smoothed density.

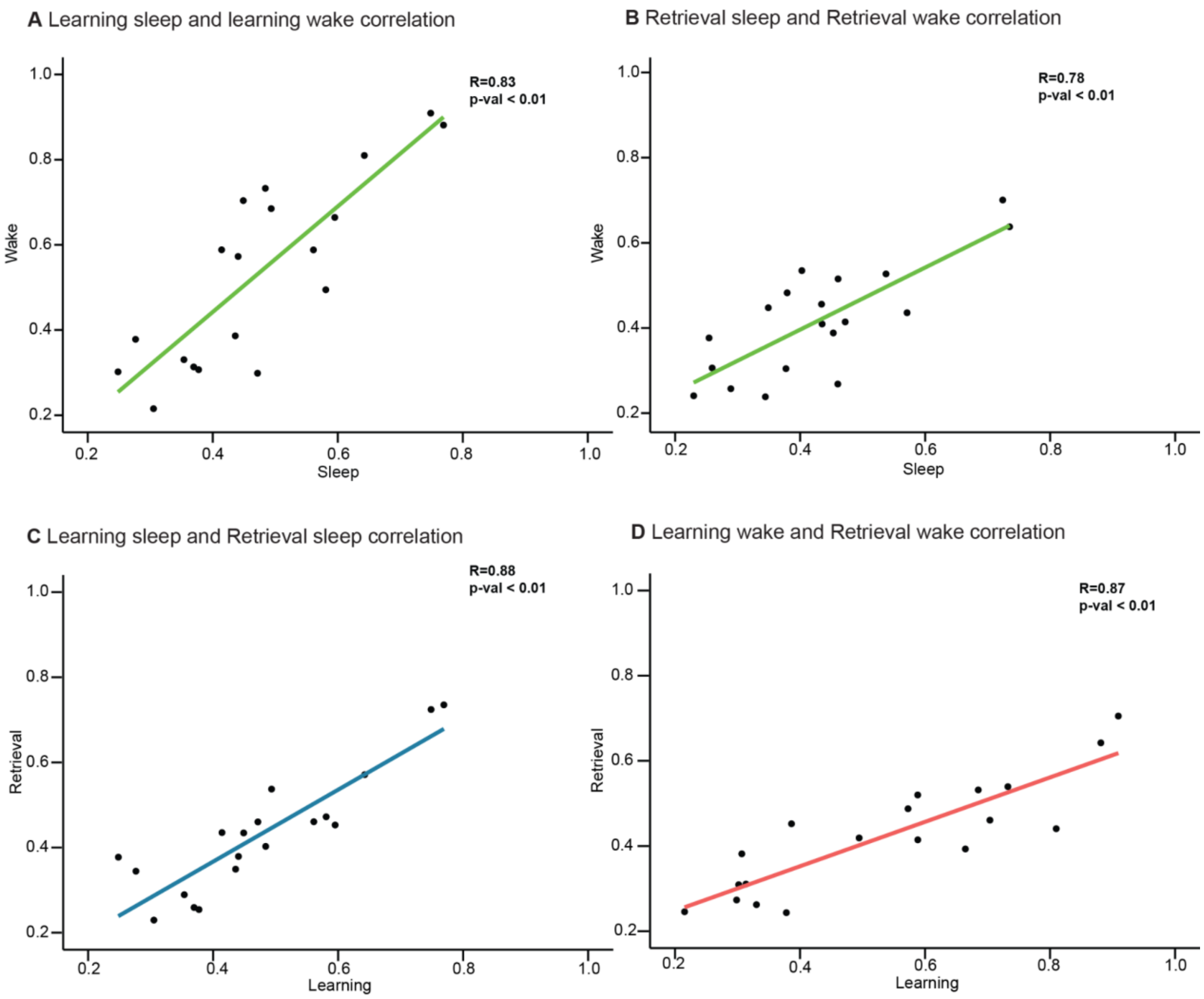

Supplementary Figure 8. Relationship between sleep and wake for A) learning and B) retrieval. Relationship between learning and retrieval for C) sleep and D) wake. Regression lines (blue – sleep, red – wake) and black dots for the individual data points are shown.
